# Drug resistance and population structure of *M.tuberculosis* isolates from prisons and communities in Ethiopia

**DOI:** 10.1186/s12879-016-2041-x

**Published:** 2016-11-21

**Authors:** Solomon Ali, Patrick Beckert, Abraham Haileamlak, Andreas Wieser, Michael Pritsch, Norbert Heinrich, Thomas Löscher, Michael Hoelscher, Stefan Niemann, Andrea Rachow

**Affiliations:** 1 College of Health sciences, Jimma University, P.O. Box 1368, Jimma city, Ethiopia; 2 Division of Infectious Diseases and Tropical Medicine, Medical Centre of the University of Munich (LMU), Munich, Germany; 3 German Center for Infection Research (DZIF), Partner Site Munich, Munich, Germany; 4 CIHLMU Center for International Health, Ludwig-Maximilians-Universität, Munich, Germany; 5 Molecular and Experimental Mycobacteriology, Research Center Borstel, Borstel, Germany; 6 German Center for Infection Research (DZIF), Partner Site Hamburg-Borstel-Lübeck, Hamburg, Germany

**Keywords:** TB genotypes, Drug resistance, TB in Ethiopia

## Abstract

**Background:**

The population structure and drug resistance pattern of *Mycobacterium tuberculosis* complex (MTBC) isolates in Ethiopian prisons and some communities is still unknown.

**Methods:**

A comparative cross sectional study was conducted on 126 MTBC strains isolated from prisons and communities in southwestern, southern and eastern Ethiopia. Phenotypic drug susceptibility testing was performed with the MGIT960 system. Combined 24-loci *Mycobacterium* interspersed repetitive unit-variable number tandem repeat and spacer oligonucleotide typing methods were used to study the MTBC population structure. The obtained data from prisons and communities were compared using statistical tests and regression analysis.

**Results:**

A diverse population structure with 11 different lineages and sub-lineages was identified. The predominant strains were the recently described Ethiopia_H37Rv like (27.52%) and Ethiopia_3 (16.51%) with equal lineage distribution between prisons and communities. 28.57% of prison strains and 31.82% of community strains shared the identical genotype with at least one other strain. The multidrug-resistance (MDR) prevalence of the community was 2.27% whereas that of prisons was 9.52%. The highest mono resistance was seen against streptomycin (15.89%).

**Conclusion:**

Tuberculosis in communities and prisons is caused by a variety of MTBC lineages with predominance of local Ethiopian lineages. The increasing prevalence of MDR MTBC strains is alarming. These findings suggest the need for new approaches for control of MDR tuberculosis in Ethiopia.

**Electronic supplementary material:**

The online version of this article (doi:10.1186/s12879-016-2041-x) contains supplementary material, which is available to authorized users.

## Background

Despite recent achievements seen in the fight against tuberculosis (TB), it still remains a significant cause of morbidity and mortality in Ethiopia [1]. This situation is worsened by an increase in prevalence of multidrug-resistant (MDR) *Mycobacterium tuberculosis* complex (MTBC) strains, defined as resistance to at least the two most powerful first line anti-tuberculosis drugs; isoniazid (INH) and rifampicin (RIF) [2].

The recent TB drug resistance survey, conducted in Ethiopia from 2011 to 2013, revealed an increase in MDR-TB with a prevalence of 2.3% and 17.8% among new and previously treated cases, respectively [3]. These data indicate that MDR-TB has been becoming a significant public health threat in the country. The occurrence and transmission of MDR-TB in confined environment like correctional facilities could further worsen the problem [4]. Due to the specific conditions found in prisons such as crowded living conditions, a large number of inmates could be infected and develop active TB disease within a short period of time. Accordingly, TB incidence and MDR rates in prisons have been found to be higher compared to that reported in the general population in several studies [5–7]. This is likely to have an impact also on the civilian population e.g., by direct transmission via visits, prison staff or later spread in community after release from prison [7, 8]. Importantly, latently infected inmates may become a reservoir of MDR-TB and a threat to the communities. In some studies performed to identify the risk factors for TB in communities previous incarceration history was described as a risk factor [9, 10].

While a number of studies confirm that TB rates are higher in prisons [7, 8, 11] little direct evidence about strain diversity and population structure of *M.tuberculosis* within prisons and the interaction of the prison setting with the community e.g., by molecular epidemiological studies is available [4, 12].

Increasing evidence suggests that the underlying genetic diversity of the MTBC has a significant impact on the pathogenicity and immunogenicity of individual strains, thus, knowledge of the regional population structure linked with phenotypic data such as with the drug resistance pattern could be relevant for the implementation of an effective TB control program tailored to specific genotypes and local circumstances [13]. Further, molecular epidemiological studies have been instrumental to define recent transmission dynamics in various settings as well as to describe the local and global population structure of the MTBC [13]. Modern molecular DNA fingerprinting methods like Mycobacterial Interspersed Repetitive Unit-Variable Number Tandem Repeats (MIRU-VNTRs) typing and spacer oligonucleotide typing (spoligotyping) are highly suited to investigate both, the population structure and transmission of the MTBC in communities or special settings such as prisons [14].

To date, no molecular epidemiological studies have been carried out to decipher MTBC strain diversity in prisons in Ethiopia. Only few studies investigated the population structure of MTBC strains in communities of central and northwestern parts of the country by spoligotyping. Due to the limited discriminatory power of this method, however, these studies reported the “ill defined” T linage as predominant strain [15–17]. There are two studies in which the combined methods of MIRU-VNTR and spoligotyping were applied, and in which Delhi/CAS was the dominating MTBC lineage [18, 19]. These two studies included only the north-western part of Ethiopia.

In order to contribute to a more comprehensive knowledge on MTBC strain diversity in the whole of Ethiopia, we conducted a pilot study to determine the population structure and drug resistance pattern of MTBC strains isolated from prisons and communities of southwestern, southern and eastern Ethiopia by combined application of MIRU-VNTR and spoligotyping. We further investigated to which extent the isolated strains were related to each other by the calculation of clustering rates.

## Methods

### MTBC strain collection at prisons and hospitals

Ethiopia is administratively organized within nine regional states and two federal cities. Oromia and Southern Nations, Nationalities and Peoples Regional State (SNNPRS) are among the three biggest regions with a total population of approximately 31,294,992 and 17,359,008 respectively [20]. Harari is the smallest regional state in Ethiopia with a population of about 210,000 [20]. Somali regional state and Dire Dawa city have a population of 5,148,989 and 387,000 respectively [20]. These four regional states and one city together cover an area where almost 65% of the total Ethiopian population resides [20]. As per Federal Ministry of Health and Health Related Indicators report of 2012/2013, TB incidence per 100,000 population in the studied regional states and city was between 258.6 (SNNPRS) and 274.7 (Dire Dawa City), respectively, per year [21].

From January 2013 to December 2013, a cross-sectional study on TB-prevalence and risk factors was conducted in 13 zonal prisons in the following regional states which are located in the Southern, South western and Eastern part of Ethiopia: Oromia, Southern Nations Nationalities and Peoples Regional State (SNNPRS) and Harari. The MTBC strains which were isolated from the sputum of symptomatic prisoners were included in this study [7]. According to the Ethiopian prison system organization, Oromia regional state had 37 (17 zonal and 20 district) prisons, while SNNPRS had 23 (13 zonal and 10 district) prisons. Harari regional state had only one zonal prison [22]. Zonal administrative prisons are the largest prisons in Ethiopian context. Most of the inmates incarcerated in zonal prisons are originated from the populations living in the respective zones. Seven out of 17 and five out of 13 prisons were selected like in a lottery from Oromia and SNNPRS, respectively, while the only prison of Harari regional state was included in the study. By this approach, ca. 35% of the total prison population of the included regional states was represented in this study. Briefly, we applied the WHO questionnaire [23] to screen up 15.495 prison inmates for the presence of TB symptoms. Two sputum samples were collected from those inmates who fulfilled the criteria for a person presumed to have TB. One sample was processed for direct smear microscopy performed at the prisons, the second sample was used for MTBC culture performed in the Jimma University Mycobacteriology laboratory. Further details on study population, methodology of sample processing and data collection as well as research outcome have been described previously by Ali et al. [7].

From August 2013 to December 2013, all MTBC strains which were routinely collected from newly diagnosed, smear positive pulmonary TB patients diagnosed in Jimma, Nekemtie, Ambo, Yabelo, Mizan, Dire Dawa, Harar and Jigjiga at ambulant health care centers or hospitals were included in this study. One early morning sputum was collected per patient and analyzed with smear microscopy in TB laboratories which are linked to hospitals in the above named cities which are located in the regional states Oromia, SNNPRS, Harari, Somali and Dire Dawa. All laboratory procedures were performed by trained hospital staff. The remaining was temporarily stored in a refrigerator until transportation to the Jimma University Mycobacteriology Laboratory. Socio-demographic and previous treatment history data were extracted from the registration book of tuberculosis clinics in respective hospitals.

### Laboratory methods

All sputum samples, from both prisoners and community based TB patients, were cultivated on LJ (BBL™ Lowenstein-Jensen Medium) at Jimma University Mycobacteriology Laboratory and afterwards transported to Research Center Borstel (RCB), Germany, for further analysis. At RCB, the strains were first reactivated on liquid mycobacterium growth indicator tube system (MGIT) 960. Drug susceptibility testing (DST) was performed using the MGIT SIRE kit at a critical concentration of streptomycin (STM) 1 μg, INH 0.1 μg, RIF 1 μg and ethambutol (EMB) 5 μg as previously described by the manufacturer [24]. DNA was extracted from all isolates for following molecular analyses, including genotyping methods [18]. Spoligotyping and 24- loci MIRU-VNTR analysis was performed as described previously [25, 26], for MIRU-VNTR typing customized kits were used (Genoscreen, Lilli, France). Spoligotypes common to more than one strain were designated as shared types (ST) and was assigned a shared international type number (SIT) according to the updated version of the international spoligotype database SpolDB4 [27]. MIRU-VNTR profiles with double alleles at a single locus were considered to represent heterogeneous populations of the same strain, whereas those with double alleles at 2 or more loci were considered to represent mixed infections or to indicate cross-contamination.

Basic strain classification and MLVA MTBC 15–9 nomenclature assignment was done using the MIRU-VNTR*plus* database [28, 29]. For the clustering analysis, samples with complete spoligotyping and MIRU-24 results were included. Cluster was defined as two or more MTBC isolates sharing identical MIRU-24 and spoligotyping patterns. Heterogeneous isolates with double alleles at only one locus were included in the cluster analysis (both patterns were compared). Isolates with no PCR amplicon at only one locus were treated as missing data at the respective locus and also were included, whereas isolates lacking amplicons at two or more loci were excluded [28, 30].

The molecular typing data was analyzed with the Bionumerics software (version 7.5; Applied Maths, Sint-Martens-Latem, Belgium) as recommended by the manufacturer. A dendrogram was generated using the unweighted pair group method with arithmetic averages (UPGMA) based on the copy number of 24-loci MIRU-VNTR. The UPGMA tree was further processed using EvolView [31].

### Statistical analysis

Data were analyzed by STATA software version 10.0. The distribution of proportions of categorical variables were compared using chi-square or Fisher’s exact test, were appropriate. Logistic regression modeling was performed to estimate the crude effect of several risk factors on clustering by comparing their association with unique isolates versus clustered isolates. Those risk factors which were significantly associated with clustering in the crude analysis were included in the multivariable regression model. *P*-values <0.05 were considered as statistically significant.

## Results

A total of 127 MTBC strains were initially isolated from sputum specimens collected in prisons (24) and hospitals (103). Out of these, 18 (14.17%) isolates were excluded from the final analysis: ten strains could not be reactivated in (liquid) culture, five isolates were mixed infections and three isolates had repeatedly inconsistent DST results.

The basic information on the 109 participants and strains included in the final analysis are depicted in Table 1. As expected, there was a significant gender difference between participants from prisons, where 90.48% were male, and hospitals with 51.14% males (*p* < 0.001). The mean age of the study subject was 29.03 (95% CI; 26.89, 31.2) with no significant difference between prison inmates and hospital participants (t = 0.93). All 88 participants from hospitals and 17 out of 21 (80.95%) inmates from prison had never received previous TB therapy. MTBC strains from prison inmates were collected in Oromia and Southern Nations, Nationalities and Peoples Regional State (SNNPRS) regional states. TB isolates from hospital patients were additionally collected in Somali regional state and Dire Dawa city administration.

**Table 1:** Basic participant information and strain characteristics

Variable	Prison, % (n/N)	Hospital, % (n/N)	*p*-value	Total% (n/N)
Sex				
Male	90.48(19/21)	51.14(45/88)	0.001	58.72(64/109)
Age				
> 45 years	14.29(3/21)	7.95(7/88)	0.366	9.17(10/109)
Previous TB treatment				
No	80.95(17/21)	100.00(88/88)	NA	
Regions				
Oromia	33.33(7/21)	10.23(9/88)		14.68(16/109)
SNNPRS	66.67(14/21)	39.77(35/88)		44.95(49/109)
Dire Dawa	–	11.36(10/88)	0.001	9.17(10/109)
Harar	–	15.91(14/88)		12.84(14/109)
Somali	–	22.70(20/88)		18.35(20/109)
Lineage				
Delhi/CAS	14.29(3/21)	17.05(15/88)		16.51(18/109)
Ethiopia_H37Rv like	42.86(9/21)	23.86(21/88)		27.52(30/109)
Euro-American Superlineage	14.29(3/21)	15.91(14/88)		15.60(17/109)
LAM	4.76(1/21)	3.41(3/88)		3.67(4/109)
Ethiopia_3	19.05(4/21)	15.91(14/88)		16.51(18/109)
Haarlem	–	9.09(8/88)	0.784	7.34(8/109)
Ural	4.76(1/21)	5.68(5/88)		5.50(6/109)
Lineage 7	–	3.41(3/88)		2.75(3/109)
EAI	–	2.27(2/88)		1.83(2/109)
X-type	–	2.27(2/88)		1.83(2/109)
Beijing	–	1.14(1/88)		0.92(1/109)
Clustering				
Yes	28.57(6/21)	31.82(28/88)	0.773	31.19(34/109)
Drug resistance				
Streptomycin^a^	20.00(4/20)^c^	14.94(13/87)	0.577	15.89(17/107)
Isoniazide	9.52(2/21)^b^	6.82(6/88)	0.669	7.34(8/109)
Rifampicin	9.52(2/21)^b^	4.55(4/88)	0.369	5.50(6/109)
Ethambutol^a^	10.00(2/20)^b^	3.41(3/88)	0.205	4.63(5/108)
MDR-resistance	9.52(2/21)^b^	2.27(2/88)	0.112	3.67(4/109)
Any drug resistance	19.05(4/21)^c^	21.59(19/88)	0.797	21.10(23/109)

N = total number of strains isolated from prisons (21) and hospitals (88)

n = number of strains in the specific subgroup

NA = not applicable

^a^note missing values

^b^includes one subject with TB in the past

^c^includes two subjects with TB in the past

### MTBC population structure

**Fig. 1:**
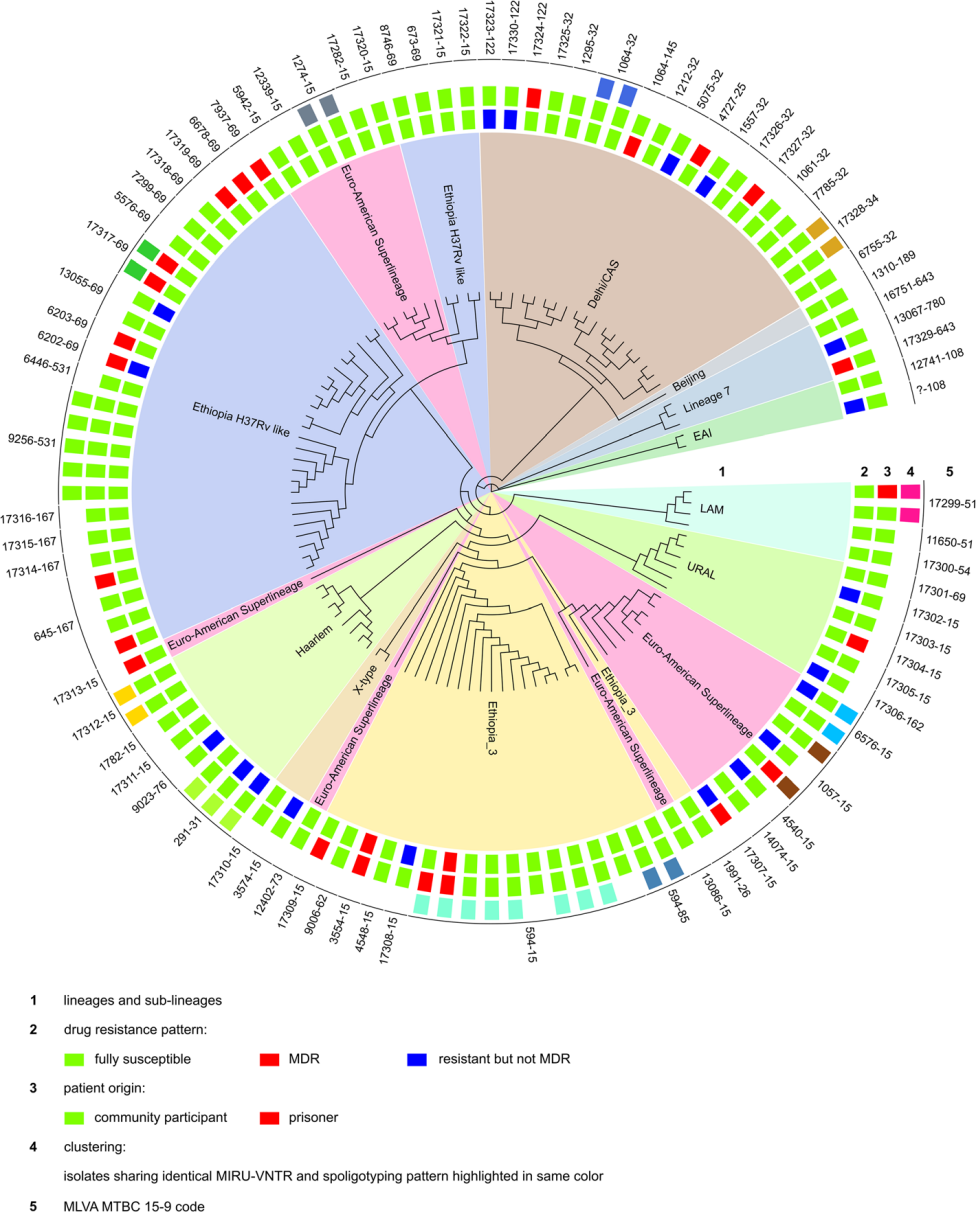
Radial UPGMA tree based on the copy numbers of MIRU-VNTR 24-loci. *Inner circle*: lineages and sub-lineages, (EAI - East African Indian, LAM - Latin American Mediterranean, CAS - Central Asia). Small rectangles in the second circle: drug resistance pattern of the individual isolates (*green* - fully susceptible, *red* - MDR, *blue* - resistant but not MDR). Small rectangles in the third circle: origin of the patient (*green* - community participant, *red* - prisoner). Small rectangle in the fourth circle: clustering according to 24-loci MIRU-VNTR and spoligotyping pattern analysis; isolates with identical genotyping profile are highlighted in same color. *Outer circle*: MLVA MTBC 15–9 code

Based on combined 24-loci MIRU-VNTR and spoligotyping patterns all 109 isolates could be classified into 11 previously described lineages and sub-lineages. The majority (27.52%) of the strains were Ethiopia_H37RV like, followed by Ethiopia_3 (16.51%) and Delhi/CAS (16.51%) (Table 1). Seventeen isolates (15.60%) were assigned to the Euro-American Superlineage. One (0.92%) strain of the Beijing lineage was found. There was no statistical difference observed between prison isolates and hospital isolates in relation to strain diversity although Haarlem, EAI, Lineage 7, X-type and Beijing lineage were not isolated in prisons (*p* = 0.784) (Table 1). The different lineages of the isolates are depicted in a radial UPGMA tree based on MIRU-VNTR analysis (Fig. 1).

The combined UPGMA tree including the Ethiopian MTBC strains of this study and those of a previous study performed by our group in the northwestern part of the country [18] is presented in the online supplement (Additional file 1: Figure S1). Interestingly, the frequency of particular genotypes is variable if different areas in the country are considered.

### Drug resistance profile

In total, 27 (24.77%) isolates were resistant to at least one anti-tuberculosis drug (Table 1, Fig. 1). The highest mono-resistance detected was against STM (15.89%), followed by INH (7.34%), RIF (5.50%) and EMB (4.63%). The overall MDR rate was 3.67% (4 out of 109). MDR-prevalence among community isolates was 2.27% (2 out of 88) whereas it was 9.52% in prison isolates (2 out of 21, with one isolate from an inmate with previous history of TB) (*p* = 0.112) (Table 1).

Among identified lineages, 1 of 3 of Lineage 7 isolates, 11.11% (2 of 18) of Ethiopia_3 isolates and 5.56% (1 of 18) of Delhi/CAS isolates were MDR TB strains (Table 2).

**Table 2:** Anti TB drug resistance and MDR pattern by lineages

Lineage (*N* = 109)	STM n (%)^b^	INH n (%)^b^	RIF n (%)^b^	EMB n (%)^b^	MDR n (%)^b^
Delhi/CAS (18)	4(23.53)	1(5.56)	1(5.56)	1(5.56)	1(5.56)
Ethiopia_H37Rvlike (30)	2(6.90)	1(3.33)	0	0	0
Euro-American Superlineage (17)	4(23.53)	0	0	0	0
LAM(4)	0	0	0	0	0
Ethiopia_3 (18)	2(11.11)	2(11.11)	3(16.67)	2(11.11)	2(11.11)
Haarlem (8)	3(37.50)	0	0	0	0
Ural(6)	0	1(16.67)	0	0	0
Lineage 7(3)	2	2	1	2	1
EAI (2)	0	0	1	0	0
X-type (2)	0	1	0	0	0
Beijing (1)	0	0	0	0	0
Total (109)^a^	17/107(15.89)	8/109(7.34)	6/109(5.50)	5/108(4.63)	4/109(3.67)

N = total number of strains

n = number of strains in the specific subgroup

^a^note missing data for STM and EMB

^b^Percentage was calculated for those lineages with more than 5 strains

### Cluster analysis

Based on 24 loci MIRU-VNTR and spoligotyping analysis 34 of 109 (31.2%) isolates were grouped into 12 different clusters ranging from 2 to 8 strains in size, with the largest cluster comprising 8 strains of the Ethiopian_3 lineage, followed by Ethiopia_H37Rv like (7 strains) and Euro-American Superlineage (6 strains) (Fig. 1 and Table 3). Among strains isolated from prisons 28.57% were clustered, which was not significantly different from the proportion (31.82%) of clustered strains collected in the community, (*p* = 0.773) (Table 1). Two strains isolated from prisoners were clustered with two strains from community members (Fig. 1). The remaining 75 isolates did not share their 24 loci MIRU-VNTR and spoligotyping patterns with any other isolate and are considered unique.

**Table 3:** Geographical information, phylogenic lineage, drug resistance pattern, and their association with strain clustering

Variables	Unique strains (n)	Clustered strains (n)	COR (95% CI)	*p*-value*	AOR (95% CI)	*p*-value^$^
Regions						
Oromia	14	2	1			
SNNPRS	32	17	3.72(0.76,18.31)	0.11	6.19 (0.92,41.83)	0.06
Dire Dawa	7	3	3.00(0.40,22.30)	0.28	3.98(0.35,44.90)	0.26
Harar	11	3	1.91(0.27,13.50)	0.52	1.36(0.15,12.70)	0.79
Somali	11	9	5.73(1.02,32.10)	0.04	8.76(1.07,71.88)	0.04
Lineage						
Delhi/CAS	14	4	1			
Ethiopia_H37Rv like	23	7	1.07(0.26,4.31)	0.93	1.20(0.28,5.20)	0.81
Euro-American Superlineage	11	6	1.91(0.43,8.48)	0.40	1.76(0.37,8.41)	0.48
LAM	2	2	3.50(0.37,33.31)	0.28	5.46(0.46,64.04)	0.18
Ethiopia_3	8	10	4.38(1.03,18.63)	0.05	8.72(1.69,45.02)	0.01
Haarlem	3	5	5.83(0.95,35.72)	0.06	7.88(0.98,63.23)	0.05
Others	14	0	-	-		
Streptomycin						
Resistant	13	4	1			
Susceptible	61	29	1.55(0.46,5.150)	0.48	NA	NA
Isoniazide						
Resistant	7	1	1			
Susceptible	68	33	3.39(0.40,28.76)	0.26	NA	NA
Rifampicin						
Resistant	5	1	1			
Susceptible	70	33	2.36(0.26,20.98)	0.44	NA	NA
Ethambutol						
Resistant	4	1	1			
Susceptible	70	33	1.89(0.20,17.54)	0.58	NA	NA
MDR						
Yes	3	1	1			
No	72	33	1.37(0.14,13.72)	0.79	NA	NA
Any resistance						
Yes	19	4	1			
No	56	30	2.54(0.79,8.16)	0.12	NA	NA

n = numbers of clustered (i.e., isolates sharing identical MIRU-24 and spoligotyping patterns with at least one other isolate) or unique (i.e., isolates that do not share their MIRU-24 and spoligotyping pattern with any other isolate) strains in each stratum

COR = crude odds ratio

AOR = adjusted odds ratio: significant variables in the crude analysis were adjusted for age, region and genotype

NA = not applicable

* = *p*-value for univariate regression model

^$^ = *p*-value for multivariate regression model

We analyzed potential risk factors for clustering in this study and found that the region from where a strain was collected and the lineage type were independently associated with clustering of TB strains (Table 3). For example the odds for clustering of TB strains from Somali region were more than eight times higher compared to those from Oromia region (Table 3). Further, lineages of Ethiopia_3 and also Haarlem were significantly associated with clustering (Table 3). In this study, drug resistance as well as demographic characteristics (age and sex) was not a risk factor for clustering.

The combined cluster analysis of the isolates collected in our current study and those of Tessema et al. showed eight clusters comprising isolates from both studies (Additional file 1: Figure S1). Those clusters were formed by strains belonging to the Delhi/CAS, Ethiopian_H37Rv like, Haarlem and Ethiopia_3 lineage.

## Discussion

In this study, we analyzed the MTBC strain population in prisons and communities of southern, southwest and eastern Ethiopia using combined spoligotyping and MIRU-VNTR typing methods. In agreement with results of previous reports from Ethiopia we could also show a high strain diversity in our study [19, 25, 32]. About 34% of the MTBC strains analyzed are Ethiopian specific Lineages and sub-lineages, Lineage 7, Ethiopia_H37Rv like and Ethiopia_3, which were described recently and have not yet been reported elsewhere [18, 19, 33]. Opposed to other studies conducted in northwest Ethiopia Delhi/CAS was not the dominating lineage [18, 19]. Tessema et al. hypothesized that the influx of Indian and Chinese peoples to Ethiopia due to growing business relations with Ethiopia introduced the Delhi/CAS lineage in the country [18]. If this hypothesis was true, the Delhi/CAS lineage dominancy might have started from the center Addis Ababa and is now processing to the periphery. This could explain why in some remote areas investigated in this study, where a relevant proportion of the population are still leading a nomadic life style which is driven by the search for water and grazing land for their cattle, not Delhi/CAS but the Ethiopian lineages are still dominating.

Clustering is a marker of recent transmission [34, 35] and knowing the clustering rate of TB strains that circulate in the community can help to evaluate the performance of TB control programs or to formulate new control strategies. The overall clustering rate in this study was 31.19% which was lower than the previously reported 45.1% from Ethiopia [18]. On the other hand our clustering rate is consistent with the 32% reported for Amhara regional state study [19]. This data could suggest that a relevant proportion of active TB cases were due to reactivation of latent infection. Indeed, there was a significant decline in TB prevalence observed in Ethiopia in the past five years [36]. This decline in active TB cases might have contributed to the lower clustering rate seen in this study compared to older reports. The cluster analysis of our strains collected in 2013 and strains analyzed by Tessema et al. in 2009 revealed several clusters, including strains from both studies, indicating that some strains remain in the population and lead either to reactivation of a remote infection or are effectively transmitted over a four years timeframe in Ethiopia [18]. Interestingly, transmission rates of active TB seemed to be higher in Somali regional state. Strains from this regional state showed a significantly higher clustering rate compared to other regional states. This finding could be explained with the geographic context of Somali regional state which is bounded by Djibouti, Somalia and Kenya [37] where a free and intensive movement of peoples living in the border areas might have contributed to an ongoing TB transmission.

As there was no statistical difference between the clustering rates in the communities (31.82%) and prisons (28.57%), our data suggest that transmission rates are not higher in prisons than in communities. This comparison, however, is hampered by the slightly different study regions, the low number of MTBC strains isolated from prison inmates and the different approaches and coverage of MTBC strain collection in prisons and communities. On the other hand we could show that MTBC strains which were isolated in prisons build clusters with strains collected from community members. This finding could indicate that TB infection was acquired in the community and developed later to active TB disease under the specific conditions found in prisons. This hypothesis is further supported by findings which were previously published by our group [7]. In that specific study, a contact with a TB case at home before incarceration was one of the strongest risk factors for active TB disease in prison [7].

Despite the great achievements seen in Ethiopia to reduce overall TB prevalence [1], the control of MDR-TB still seems a distant prospect. In this study we have observed an MDR-TB prevalence in the community of 2.27% which was consistent with the 2.3% of the Ethiopian public health institute (EPHI) survey report performed in year 2014 [3]. However, comparing our findings with 0.8% seen in 2002 [38] and the 1.6% estimation of WHO in 2011 indicates that MDR-TB is increasing with alarming rate through time [3, 39]. In prisons, the MDR TB prevalence (9.52%) and also the number of subjects with TB in the past was higher than in communities, although, this difference was not statistically significant in our analysis of a limited number of isolates. This result is alarming since prisons may act as a reservoir for MDR-TB in the country. The occurrence of a single MDR-TB case in prison might have huge implications for prison health and the community, considering bidirectional communication between both.

This study has several limitations and the findings should be interpreted with care. First, due to a relative short study period, the number of MTBC isolates in this study, specifically the number of strains from prisoners, is lower than in other reports [18, 19]. Therefore, the magnitude of clustering and also specific risk factors on clustering, e.g., drug resistance, could have been undetected or underestimated. Second, only TB strains from selected communities (which had access to a TB laboratory located in neighboring hospitals) and prisons (to which the investigators had permission to enter) were included in the analysis. This fact could introduce selection bias, especially as only characteristics of TB strains from participants who had access to the health system or who were inmates in the rather huge zonal prisons could be studied. Third, clustering rates could be imprecise and rather reflecting the spread of dominant strains types but not recent transmission as not all MTBC strains in the study areas were analyzed but only those collected in the catchment area of large hospitals and large prisons. Fourth, the rather cross-sectional study approach in both prison and community settings did not allow for the investigation of risk factors of TB transmission in cases with an incubation period of more than 12 or five months, respectively. Fifth, HIV test results were not available for community based patients. This hampered the analysis of the influence of HIV on TB transmission and clustering. Finally, this pilot study highlights the need to further investigate the drug resistance, population structure and transmission dynamics of TB in communities and prisons in Ethiopia as well as the interaction of both groups in a larger and prospective survey.

## Conclusion

Our study provides first data on MTBC population structure and drug resistance pattern of strains found in Ethiopian prisons and in regional states of the country which were not studied before. Our findings suggest that TB is still not sufficiently controlled in specific, potentially remote, areas of the country and highlight the need for improved tools and new strategies aiming for MDR-TB control, especially in prisons. The fact that TB strains from prisoners are forming clusters with community based TB strains is worth noting and stresses the importance of the inclusion of prisons in strategies for TB control in the whole of Ethiopia. Future studies of sufficient duration and area-wide strain collection need to be performed to improve our knowledge on risk factors for TB transmission in Ethiopia.

## Additional file

Additional file 1: Figure S1. Radial UPGMA tree based on the copy numbers of MIRU-VNTR 24-loci of 109 isolates of the current study and additional 240 isolates from Tessema et al. Inner circle: lineages and sub-lineages (EAI - East African Indian, LAM - Latin American Mediterranean, CAS - Central Asia). Small rectangle in the second circle: affiliation of the isolate (green - current study, red - Tessema et al. northwest Ethiopia). Small rectangle in the third circle: drug resistance pattern (green - fully susceptible, red - MDR, blue - resistant but not MDR). Small rectangle in the outer circle: clustering according to 24-loci MIRU-VNTR and spoligotyping pattern analysis; isolates with identical genotyping profile are highlighted in same color. (PNG 2468 kb)

## Abbreviations

DST: Drug susceptibility testing
EMB: Ethambutol
INH: Isoniazide
MDR: Multi-drug resistance
MDR-TB: Multi drug resistance tuberculosis
MGIT960 system: *Mycobacterium* growth indicator tube system
MIRU-VNTRs: Mycobacterial interspersed repetitive unit-variable number tandem repeats
MLVA: Multiple locus variable number tandem repeat analysis
MTBC: *Mycobacterium tuberculosis* complex
RIF: Rifampicin
SIT: Shared international type number
SNNPRS: Southern Nations Nationalities and Peoples Regional State
STM: Streptomycin
TB: Tuberculosis
UPGMA: Unweighted pair group method with arithmetic averages
