# Supplementary figures and images for: Drug resistance and population structure of M.tuberculosis isolates from prisons and communities in Ethiopia
Source: BMC Infect Dis. 2016 Nov 21;16:687. doi: 10.1186/s12879-016-2041-x (PMC5117695; doi:10.1186/s12879-016-2041-x)

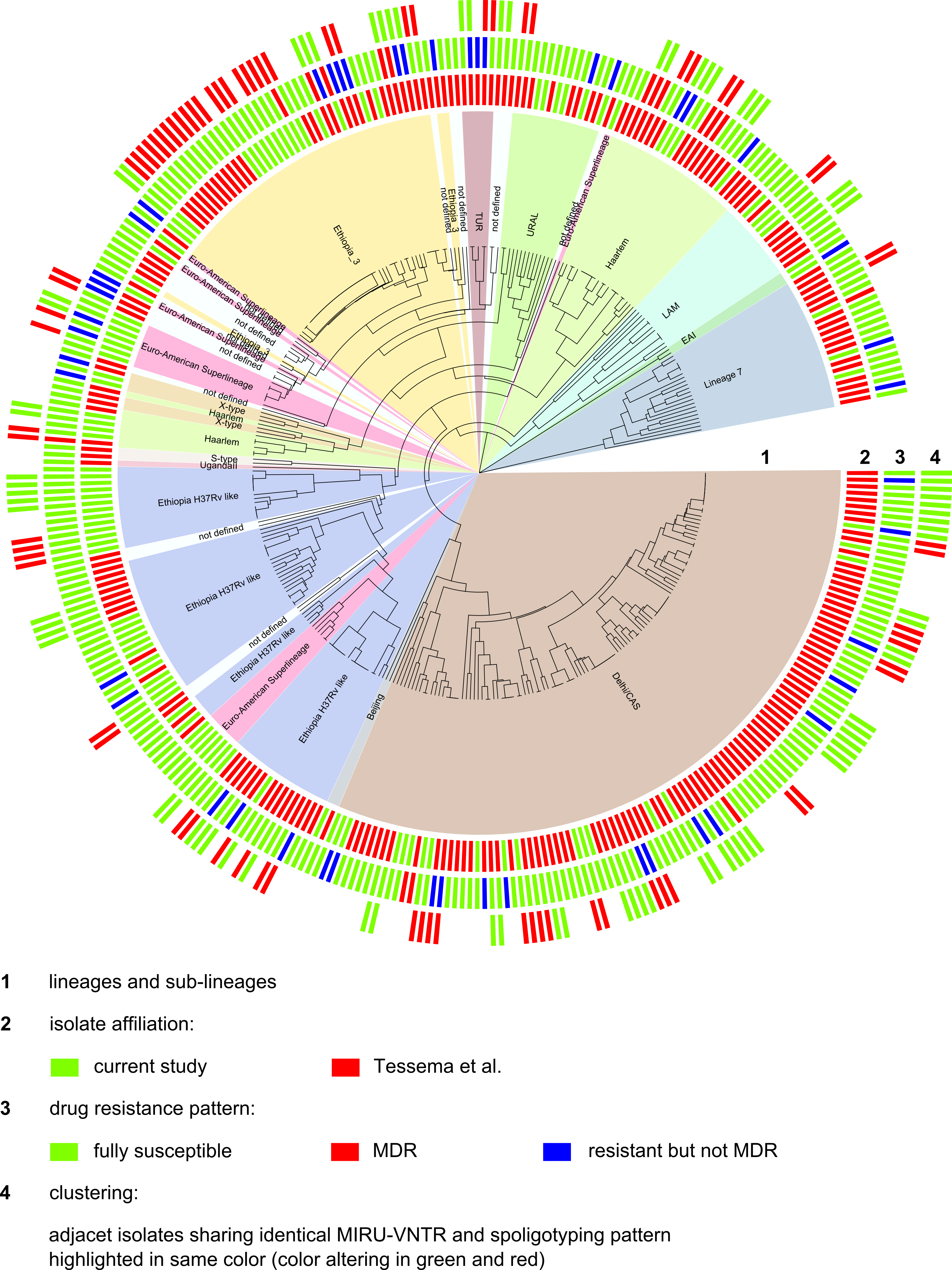

Supplement: Additional file 1: Figure S1. — Radial UPGMA tree based on the copy numbers of MIRU-VNTR 24-loci of 109 isolates of the current study and additional 240 isolates from Tessema et al. Inner circle: lineages and sub-lineages (EAI - East African Indian, LAM - Latin American Mediterranean, CAS - Central Asia). Small rectangle in the second circle: affiliation of the isolate (green - current study, red - Tessema et al. northwest Ethiopia). Small rectangle in the third circle: drug resistance pattern (green - fully susceptible, red - MDR, blue - resistant but not MDR). Small rectangle in the outer circle: clustering according to 24-loci MIRU-VNTR and spoligotyping pattern analysis; isolates with identical genotyping profile are highlighted in same color. (PNG 2468 kb) [file 12879_2016_2041_MOESM1_ESM.png]
